# The MCIB Model: A Novel Theory for Describing the Spatial Heterogeneity of the Tumor Microenvironment

**DOI:** 10.3390/ijms251910486

**Published:** 2024-09-29

**Authors:** Minghao Guo, Yinan Sun, Xiaohui Wang, Zikun Wang, Xun Yuan, Xinyi Chen, Xianglin Yuan, Lu Wang

**Affiliations:** 1 Department of Oncology, Tongji Hospital, Tongji Medical College, Huazhong University of Science and Technology, Wuhan 430030, China; u202110355@hust.edu.cn (M.G.); yuanxun@tjh.tjmu.edu.cn (X.Y.); cxyjozee@foxmail.com (X.C.); 2 Tongji Medical College, Huazhong University of Science and Technology, Wuhan 430030, China; sunyinan@hust.edu.cn (Y.S.);; 3 The Center for Biomedical Research, Department of Respiratory and Critical Care Medicine, NHC Key Laboratory of Respiratory Diseases, Tongji Hospital, Tongji Medical College, Huazhong University of Science and Technology, Wuhan 430030, China; d202282145@hust.edu.cn

**Keywords:** spatial heterogeneity, TME, MCIB model, tumor targeted therapy

## Abstract

The tumor microenvironment (TME) can be regarded as a complex and dynamic microecosystem generated by the interactions of tumor cells, interstitial cells, the extracellular matrix, and their products and plays an important role in the occurrence, progression and metastasis of tumors. In a previous study, we constructed an IEO model (prI-, prE-, and pOst-metastatic niche) according to the chronological sequence of TME development. In this paper, to fill the theoretical gap in spatial heterogeneity in the TME, we defined an MCIB model (Metabolic, Circulatory, Immune, and microBial microenvironment). The MCIB model divides the TME into four subtypes that interact with each other in terms of mechanism, corresponding to the four major links of metabolic reprogramming, vascular remodeling, immune response, and microbial action, providing a new way to assess the TME. The combination of the MCIB model and IEO model comprehensively depicts the spatiotemporal evolution of the TME and can provide a theoretical basis for the combination of clinical targeted therapy, immunotherapy, and other comprehensive treatment modalities for tumors according to the combination and crosstalk of different subtypes in the MCIB model and provide a powerful research paradigm for tumor drug-resistance mechanisms and tumor biological behavior.

## 1. Introduction

With the rapid development of single-cell sequencing, spatial transcriptomics, and metabolomics, the unique and dynamic tumor microenvironment (TME), which is composed of tumor cells and various surrounding mesenchymal cells, cellular metabolites, cytokines, and the extracellular matrix (ECM), has been fully researched. In the process of tumor growth and evolution, tumor cells have always faced tremendous selection pressure, including drugs and anti-tumor immunity. On the one hand, tumor cells increase their heterogeneity through the behaviors described in the “cancer stem cell theory” and “cloning evolutionary theory” to strive for more survival opportunities for the cancer cell population, and on the other hand, they improve the cell state with the help of stromal cells, blood supply, and cytokines in the TME, making them the key factors that determine the fate of the cancer cell population in the body [1]. In fact, the main role of medical oncology therapy today can be summarized as the direct killing of tumor cells (e.g., traditional chemotherapy drugs that interfere with nucleic acid metabolism as the main mechanism) and alteration of the tumor microenvironment (e.g., immunotherapy and anti-angiogenic targeted drugs). Therefore, the theoretical and basic research of TME has led to the discovery of more new targets and new biomarkers within TME [2]. TME will remain one of the most important topics in oncology research for the foreseeable future. Over the past 30 years, with the establishment of multiple large-scale biological databases, advances in sequencing technologies, imaging technologies, and structural biology, in-depth characterization of TME has been made possible, especially for the tumor immune microenvironment (TIME). Researchers have developed a number of mathematical modeling methods to describe and predict TIME [3,4]. However, with the rapid development of artificial intelligence (AI) and big data science, TME research has not yet shown a tendency to transform into analytic intelligent big data models for the entire TME landscape. TME not only includes various types of non-tumor cells in tumor tissues, but also covers a variety of cellular biological effects such as energy metabolism, substance metabolism, signal crosstalk, immune effect, microbial action, and other cellular biological effects at various stages of tumorigenesis and development, thus forming a multi-level and three-dimensional dynamic interaction network [2,5,6]. As mentioned above, while TME research is extremely important in oncology research, its vast scope of research makes it easy to overlook the systematic and holistic nature that is required for highly complex TME studies.

Therefore, we propose a simplified model for the systematic improvement of TME research; that is, according to the heterogeneity of TME in the spatiotemporal evolution, the components and change processes of the spatial and temporal dimensions are analyzed in the form of TME subsets and named after the combination of initials. The interactions between TME subsets are presented in detail in the discussion section. Such a model is not only a regular summary of the existing TME research, but also a necessary preparation and theoretical framework for the progress of TME research towards comprehensive and systematic progress. In our previous study, we constructed an IEO model according to the chronological order of hepatic microenvironment development and classified the hepatic metastatic microenvironment into prI-, prE-, and pOst-metastatic niches [7]. However, the spatial composition of the TME at each stage has yet to be explored. To fill in the gaps that remain to be explored in the spatial composition of TME at each stage, we classified the TME into the TMME, TCME, TIME, and TBME, which together constitute the main components of the MCIB model according to the different cell types, metabolites, and biomarkers in the TME and according to the different directions of the clinical treatment modalities, as shown in Table 1. The presentation of the MCIB model parses the whole TME landscape from different spatial functional structures and therapeutic directions for different microenvironments.

## 2. The Characteristics of the MCIB Model

**Figure 1:**
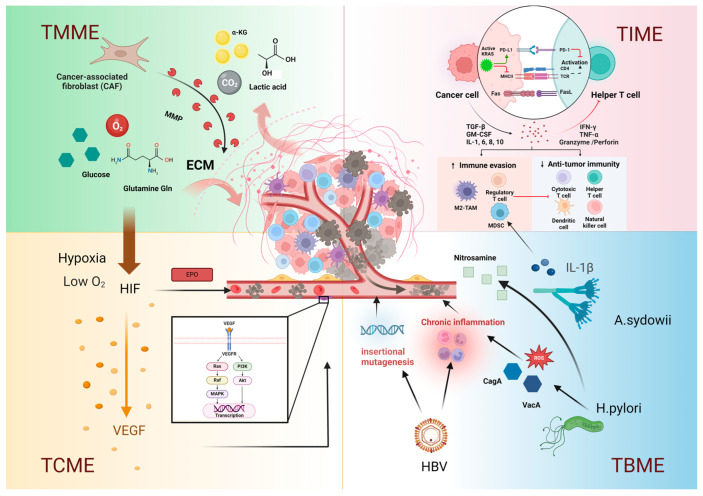
Composition of the MCIB: the description of the different microenvironment subtypes of the MCIB. TMME, tumor metabolic microenvironment; TCME, tumor circulatory microenvironment; TIME, tumor immune microenvironment; TBME, tumor microbiota microenvironment; MMP, matrix metalloproteinase; ECM, extracellular matrix; α-KG, α-ketoglutarate; HIF, hypoxia-inducible factor; VEGF, vascular endothelial growth factor; VEGFR, vascular endothelial growth factor receptor; EPO, erythropoietin; PD-1, programmed death-1; PD-L1, programmed death ligand-1; MHC, major histocompatibility complex; TAM, tumor-associated macrophage; MDSC, myeloid-derived suppressor cell; TCR, T cell receptor; Fas, factor-related apoptosis; FasL, factor-related apoptosis ligand; ROS, reactive oxygen species; VacA, vacuolating cytotoxin A; CagA, cytotoxin-associated gene A; HBV, hepatitis B virus. Figure created with BioRender.com (accessed on 31 January 2024).

The spatial components of the TME are not bystanders in tumorigenesis and progression but rather act as inhibitors or promoters of tumor growth, invasion, and metastasis [5]. In the early stages of tumor progression, due to the special metabolic pathways and rapid proliferation of tumor cells, a tumor metabolic microenvironment (TMME) consisting of various metabolites, the ECM, and a relatively hypoxic microenvironment is formed around tumor cells. The hypoxic microenvironment, as well as cellular metabolites, stimulate stromal cells to undergo tumor angiogenesis and form the tumor circulating microenvironment (TCME), which is critical for tumor metastasis [8]. Tumor cells are antigenic, but they can also evade recognition and destruction by antitumor immune cells. Some tumor-promoting immune cells in the TME can secrete tumor-promoting regulatory molecules and inhibit the activation of antitumor immune cells, which leads to the gradual formation of an immunosuppressive tumor immune microenvironment (TIME) [9]. A variety of cancer biomarkers can also interact with TIME. Finally, the tumor-associated microbiota is an important component of the TME and plays a key role in tumorigenesis, progression, metastasis, and immune regulation; this process is called the tumor microbiota microenvironment (TBME). Figure 1 provides an overview of the composition of the MCIB groups, including four subtypes of the TME and their components, as presented in detail in the following sections. For about half a century, in the field of clinical oncology, in addition to traditional surgery, radiotherapy, and chemotherapy, new therapeutic approaches for cancer targeting the four components of the TME have also been rapidly developed, such as pharmacological hormone therapies, tumor targeted therapy, photodynamic therapy, antibody drug conjugates therapy, radionuclide therapy, oncolytic virus therapy, immune checkpoint inhibitors, and adoptive cell therapy [10]. These therapies, which cover a multi-dimensional, multidisciplinary body of knowledge, can be summarized into metabolic interference targeted therapy, antiangiogenic targeted therapy, immunotherapy, and biotherapy, which proves the scientific and practical nature of the MCIB model.

## 3. Tumor Metabolic Microenvironment

The metabolic reprogramming of tumor cells and tumor-associated mesenchymal stromal cells is the fundamental reason why these cells create an MME that is different from that of normal tissues. Research on tumor metabolism began with the finding of Otto Warburg that tumors consume abundant glucose and secrete lactate even in the presence of oxygen, a phenomenon known as the Warburg effect or aerobic glycolysis [11]. Tumor cells and tumor-related stromal cells change metabolites in the microenvironment (such as glucose, lactic acid, amino acids, oxygen, carbon dioxide, etc.) through metabolic pathways different from those of normal tissue cells (such as aerobic glycolysis, “glutamine addiction”, and the reverse “Warburg effect”). The TME is characterized by low pH, hypoxia, and nutrient deficiency, and the use of metabolites in the microenvironment accelerates tumor growth and proliferation. The TMME model provides a platform for tumor and stromal cells to interact with each other and contributes to the metabolic interaction between tumor cells and cells in the microenvironment.

### 3.1. The Metabolic Pathways of Tumor Cells

Tumor cells function differently from normal tissue cells and have significantly faster growth and proliferation rates; therefore, these cells must be able to adjust their metabolic pathways according to the environment to meet the need for bioenergy and biosynthesis. Aerobic glycolysis is the most typical metabolic hallmark of tumor cells. In addition, tumors obtain more glucose by upregulating the high-affinity glucose transporter protein GLUT1, downregulating transporter proteins with low affinity, consuming large amounts of glucose, converting large amounts of glucose-derived pyruvate to lactate through lactate dehydrogenase (LDHA) activity, and facilitating the transfer of lactate from tumor cells to the extracellular compartment by monocarboxylic acid transporter proteins (MCTs), even in oxygen-rich environments [12,13,14]. Although aerobic glycolysis produces ATP much less efficiently than oxidative phosphorylation does, the rapid increase in energy supply is better suited to the rapid proliferation of tumor cells [15]. Lactate, the end product of aerobic glycolysis, promotes NAD+ regeneration in the intracellular compartment, which in turn maintains glycolysis [6]. Intermediate products are used as raw materials for other metabolic reactions to synthesize biological substances and maintain the balance of electrical potential and redox reactions.

Since a large amount of pyruvate is used to generate lactate, researchers have generally concluded that uncoupling of glycolysis from the tricarboxylic acid (TCA) cycle is achieved in tumor cells [16,17,18]. The TCA cycle occupies a central position in cellular metabolism, and tumor cells remain dependent on the tricarboxylic acid cycle for some energy production and biosynthesis. Influenced by the high metabolism of tumor cells and abnormal vascular structure, the TME as a whole is hypoxic, which triggers an increase in the expression of hypoxia-inducible factors (HIFs) [19]. As a class of transcription factors, HIFs not only promote the expression of erythropoietin (EPO) and angiogenesis but also mediate the activation of the pyruvate dehydrogenase kinase-1 gene to phosphorylate and inactivate pyruvate dehydrogenase in the TCA cycle, thereby reducing the flux of glucose-pyruvate-acetyl coenzyme A into the TCA cycle [19,20]. Therefore, tumor cells are more dependent on glutamine for fuel delivery to the TCA cycle than are normal cells [21,22].

Increased catabolic utilization of glutamine is also considered an important marker of metabolic reprogramming in tumor cells. Glutamine is first hydrolyzed by glutaminase (GLS) to generate glutamate, which is then dehydrogenated by glutamate dehydrogenase (GLUD) to generate α-ketoglutarate (α-KG), which enters the TCA cycle to replenish biological raw materials that have been reduced due to aerobic glycolysis or serve as a cosubstrate for glutamic-oxaloacetic transaminase (GOT) and glutamate pyruvate transaminase (GPT) to generate alanine and aspartic acid, respectively, which are then converted by alanine or aspartate transaminases (TAs) to α-KG [21,23]. Both α-KG and ammonia, metabolic byproducts of glutamine, can serve as raw materials for the synthesis of purines and pyrimidines, sustaining the rapid proliferation of tumor cells [24,25]. Tumor cells increase GLS and glutamine transporter levels, enabling tumor cells to acquire large amounts of energy and biomacromolecules through glutamine catabolism, leading to glutamine addiction in various cancer types, including myeloma and glioma [21].

### 3.2. Tumor Extracellular Matrix

As an important noncellular component of the TME, the ECM is closely related to tumor proliferation, metastasis, and immunosuppression. The ECM of normal cells is mainly composed of collagen, noncollagens, elastin, proteoglycans, and glycosaminoglycan, as well as soluble cytokines, such as growth factors and cytokines [26]. The ECM can participate in maintaining cell morphology, polarity, and cellular homeostasis by interacting with cell surface receptors and mediating the diffusion of signaling molecules [27]. The ECM of tumor cells is mainly produced by cancer-associated fibroblasts (CAFs), which are characterized by increased synthesis and deposition of collagen fibers and increased stiffness (also described as ECM stiffness); these processes are usually accompanied by high expression of ECM remodeling enzymes and disruption of the connection to the basement membrane [26,28].

ECM remodeling enzymes are key factors in the production of the tumor extracellular matrix, among which matrix metalloproteinases (MMPs) play the most prominent role. The MMP in the TME is mainly derived from CAFs and immune cells with pro-promoting effects, such as myeloid-derived suppressor cells (MDSCs), neutrophils, and M2 macrophages [29]. MMPs are a family of zinc-dependent endopeptidases that extensively bind and digest various types of ECM proteins or selectively release cell-surface-bound cytokines, growth factors, or their receptors, thereby interfering with ECM integrity, immune cell recruitment, and tissue renewal and disrupting normal cell function [30,31]. For example, MMP-14 can induce collagen I, II, and III in the tumor extracellular matrix, and MMP-2 and MMP-9 can degrade collagen IV, thus mediating tumor cell invasion into the basement membrane and inducing tumor invasion and metastasis [32,33]. In addition, the hypoxic microenvironment caused by the abnormal metabolism of tumor cells can induce increased expression levels of several ECM remodeling enzymes (such as LOX and collagen prolyl 4-hydroxylase (C-P4H)), which mediate the modification of collagen and thus participate in ECM remodeling [34]. MMP-2, MMP-9, and MMP-14 can be upregulated in some types of tumor cells through a hypoxia-inducible factor (HIF)-dependent mechanism [35]. In addition, CAFs, as producers of MMPs, are important contributors to structural and functional remodeling of the tumor extracellular matrix, but hypoxia can inhibit tumor ECM remodeling induced by CAFs through PHD-2 regulation [36,37]. In addition to the hypoxic microenvironment, the acidic microenvironment is also the result of the regulation of TMME [6]. Voltage-gated sodium channels (VGSCs), as an important cancer biomarker, can promote the reduction of extracellular matrix pH, which promotes the degradation of extracellular matrix by cysteine cathepsin, thereby promoting tumor invasion and metastasis [38].

### 3.3. Metabolic Interactions between Tumor Cells and CAFs in the TME

In 1889, Paget proposed the classical “seed and soil” theory, which states that stromal cells play an important role in the development of tumors [39]. As the most important class of stromal cells, CAFs are the most abundant cellular component in the TME, and their metabolic crosstalk is closely related to the metabolic reprogramming of tumor cells [40,41].

The Warburg effect is a marker of metabolic remodeling in tumor cells, and the reverse “Warburg effect” occurs in CAFs; that is, compared with that in normal fibroblasts, the glucose metabolism in CAFs is significantly altered from oxidative phosphorylation to aerobic glycolysis [42]. CAFs produce and secrete metabolites such as pyruvate, lactate and glutamine, which serve as nutrients to support the metabolic needs of tumor cells, enabling their metabolic coupling [43]. Loss of stromal caveolin-1 (Cav-1) in CAFs was significantly associated with recurrence, metastasis, drug resistance, and poor clinical outcome in breast cancer patients [44]. This finding may be because Cav-1-deficient fibroblasts upregulate the expression of glycolytic enzymes, thus providing tumor cells with energy-rich metabolites through paracrine signaling to promote tumor angiogenesis and growth [45]. Another report on breast cancer indicated that the promoters of the rate-limiting glycolytic genes FBP1, PKM and LDHA were specifically methylated in CAFs, where PKM and LDHA expression levels increased, thereby increasing glycolysis in CAFs [46]. Moreover, CAFs can synthesize the amino acids required for tumor cells through the TCA cycle. The Ras inhibitor RASAL3 showed epigenetic silencing in human prostate CAFs, which can lead to oncogenic Ras activity to drive glutamine synthesis mediated by macropinocytosis for uptake by prostate cancer cells, increased flux through the TCA cycle, and increased ATP generation and prostate cancer cell growth [47]. Studies have shown that aspartate and glutamate are exchanged between CAFs and head and neck squamous cell carcinoma (HNSCC) cells via the aspartate/glutamate transporter solute carrier family 1 membrane 3 (SLC1A3) channel [48]. Aspartate provides materials for nucleotide biosynthesis in tumor cells, and glutamate in CAFs provides materials for glutathione synthesis pathways [48].

## 4. Tumor Circulatory Microenvironment

The role of tumor neovascularization in tumor nutritional support and metastasis has been widely validated. Endothelial cells, red blood cells, white blood cells, platelets, circulating tumor cells, and plasma components, together with tumor tissue, form the tumor circulatory microenvironment (TCME).

### 4.1. Tumor Angiogenesis and Antiangiogenic Drugs

In 1971, Folkman first proposed that angiogenesis is a necessary condition for the development and growth of solid tumors larger than 1–2 mm^3^ [49]. Expanded tumor growth and corresponding tumor angiogenesis are multistep, complex biological processes that are positively or negatively regulated by multiple sets of regulatory factors. Many studies and reviews have detailed the mechanism of tumor angiogenesis. Generally, there are two main mechanisms of tumor angiogenesis: (i) Sprouting angiogenesis: under the regulation of vascular endothelial growth factor (VEGF), the tip cells of the vascular endothelium migrate along the chemotactic path, and to secrete matrix-degrading proteins, stalk cells are subsequently separated and form a new vascular lumen [50,51]. (ii) Vascular mimicry: in the absence of endothelial cell involvement, tumor cells undergo external stimuli to form tubular structures that can accommodate the passage of blood, but the underlying mechanism is unclear [52,53]. VEGF family members are the most important regulatory molecules involved in tumor angiogenesis. They activate the mitogen-activated protein kinase (MAPK) pathway and phosphoinositide 3 kinase (PI3K)/Akt pathway in vascular endothelial cells, thereby promoting the metabolism, proliferation, growth, and differentiation of vascular endothelial cells [54]. In addition, the hypoxic TME favors tumor angiogenesis. Some of the subunits of HIF highly expressed in the hypoxic TME have been shown to be directly bound to the promoter to increase the expression levels of VEGF and VEGF receptors (VEGFRs) and thus promote angiogenesis [8,55]. Other regulators in the TME, such as fibroblast growth factor-2 (FGF2) and the platelet-derived growth factor (PDGF) family, can also participate in the generation of tumor vasculature [50]. The former can activate the degradation of the extracellular matrix of vascular endothelial cells and has synergistic effects on VEGF, while the latter can promote vascular maturation and pericyte recruitment and induce the upregulation of VEGF [56,57].

Antiangiogenic therapy is an important targeted therapy for cancer. The main principle of antiangiogenic drugs is to act on the targets of angiogenesis, interfere with the key signaling pathways of protumor angiogenesis, downregulate the expression of proangiogenic factors, inhibit tumor angiogenesis, and achieve the effect of “starving” tumors [58,59]. Unlike usual tumor-targeted therapies, antiangiogenic agents do not require that patients test positive for driver genes and do not need genetic testing for use. Current antiangiogenic drugs include small-molecule multitarget angiogenesis inhibitors, macromolecule single-target angiogenesis inhibitors, and endogenous pan-target angiogenesis inhibitors. Since VEGF is considered to be the most important regulator of tumor angiogenesis, antibodies against VEGF and VEGFR, as well as small molecule tyrosine kinase inhibitors (TKIs) that can inhibit VEGF receptor signaling, are the main types of antiangiogenic therapy [60].

### 4.2. Hypoxic Microenvironment

The median oxygenation level within untreated tumor tissue is approximately 0.3% to 4.2%, which is much lower than the 3% to 7.4% in normal tissues [61]. This tumor oxygen microenvironment is the result of uncontrolled rapid tumor proliferation and the abnormal structure of tumor vessels. To adapt to hypoxia, tissue cells can increase the expression of HIF [62]. As a hypoxia-induced transcription factor, HIF can regulate the expression of various colony-stimulating factors and growth factors, such as the EPO gene and VEGF gene [19,63]. Although the oxygen microenvironment of tumors can promote angiogenesis, unlike vascular endothelial cells in normal tissues, tumor-associated endothelial cells (TECs) are irregular in size and shape and have large intercellular holes and abnormal sprouts, leading to irregular vascular diameter and progression and nonlaminar blood flow [64,65]. Relatively disordered and irregular vascular growth makes it difficult to deliver enough oxygen to tumor tissue, and this process fails to disrupt the imbalance between increased oxygen consumption and insufficient oxygen supply, allowing the TME to maintain a relatively hypoxic state.

### 4.3. Effect of Tumor Blood Vessels on Tumor Metastasis

Tumor metastasis is the leading cause of death in cancer patients, and blood tract metastasis is one of the main routes of malignant tumor metastasis. Structurally abnormal tumors have high neovascular permeability and are more prone to leakage [66,67]. Therefore, tumor cells are more likely to penetrate tumor blood vessels and enter systemic blood circulation, becoming circulating tumor cells. In addition, TECs play an important role in tumor metastasis. CXCR7, which is highly expressed in TECs, plays a key role in transendothelial migration mediated by CXCL12/CXCR4 in CXCR4 (+)CXCR7 (+) human tumor cells [68,69].

## 5. Tumor Immune Microenvironment

Tumor tissues always show infiltration of immune cells and immunoreactive substances in the body, as well as surveillance of the immune system. The body’s immune response to tumors, immune evasion of tumors, and competition between tumor cells and immune cells in terms of metabolism and nutrition together constitute the tumor immune microenvironment (TIME).

The effect of immune cells on tumors manifests in two directions: antitumor and protumor effects. Antitumor cells usually include effector T cells and natural killer (NK) cells, while protumor cells mainly include regulatory T cells (Tregs), MDSCs, and tumor-associated macrophages (TAMs) [70,71]. However, the role of B cells in tumor immunity is still somewhat controversial [72]. Antitumor and protumor immune cells work together to construct a tumor immune microenvironment to regulate tumor sensitivity to immune clearance. In recent years, many studies have confirmed the good inhibitory and killing effects of immunotherapy on tumor cells, as well as the substantial changes in the immune microenvironment, but the mechanism of the tumor immune microenvironment needs to be further elucidated [70,73].

### 5.1. Innate Immune Cells in the TIME

Macrophages are one of several important classes of immune cells that are recruited to tumor sites. TAMs are generally divided into two subtypes: classically activated macrophages (M1) and alternatively activated macrophages (M2). The activation of M1-like TAMs is mediated by cytokines such as IFN-γ and IL-2 [74]. M1-like TAMs can secrete TNF-α and IL-1 and possess powerful antigen-presenting ability [75]. As the main cell subset of tumor-infiltrating TAMs, M2-like TAMs are activated by IL-4, IL-6, and IL-13 and express immunosuppressive cytokines such as TGF-β, IL-4, IL-10, IL-13, and IL-1 receptor antagonists, which mainly induce and participate in the Th2 immune response and are protumor immune cells [76,77,78].

NK cells act as innate lymphocytes (ILCs) that induce target cell lysis [79] by releasing perforin and granzyme after binding to target cells. NK cells also release inflammatory factors such as IFN-γ, TNF-α, and chemokines (CCL3, CCL4, and CCL5) to increase their antitumor activity [80]. In addition, NK cell-mediated cytotoxic effects can induce significant CD8^+^ T cell responses, thereby enhancing antitumor activity [81].

MDSCs are a heterogeneous myeloid cell population consisting of pathologically activated immunosuppressive monocytes and neutrophils [5]. MDSCs secrete immunosuppressive cytokines such as IL-10 and TGF-β; inhibit effector T cells, natural killer cells, and dendritic cell (DC)-mediated immune responses; and promote the expansion and induction of Treg cells [82,83].

Dendritic cells (DCs) are full-time antigen-presenting cells (APCs) that exert antitumor effects by presenting antigenic information from the TME to adaptive immune cells [5].

### 5.2. Adaptive Immune Cells in the TIME

T cells are the most important tumor immune cells. CD8^+^ T cells can recognize major histocompatibility complex (MHC) class I molecules and secrete IFN-γ and TNF-α to exert antitumor effects, and cytotoxic T cells (CTLs) can also directly exert cytotoxic effects by releasing perforin and granzyme or inducing FasL-mediated apoptosis [84]. CD4^+^ T cells can present tumor antigen epitopes; promote the activation of CD8^+^ T cells and the effector and memory functions of CTLs; and secrete IFN-γ, IL-2, and TNF-α to improve CTL activity and assist in killing tumor cells [85]. Tregs play an important role in reducing the immune response toward autoantigens and suppressing immune responses that are harmful to the body [86]. Tregs can express IL-2 receptors as well as immunosuppressive cytokines such as CTLA-4, IL-10, IL-35, and TGF-β and inhibit the proliferation and activation of effector T cells and NK cells, thereby inhibiting antitumor immunity [87,88].

However, the role of B cells in tumors is controversial. On the one hand, B cells can exert limited antitumor effects [5] through complement-dependent cytotoxicity (CDC) and antibody-dependent cell-mediated cytotoxicity (ADCC). On the other hand, B cells can also support tumor cell growth, proliferation, and metastasis by secreting protumorigenic factors [89].

### 5.3. Immunotherapy and Screening for Cancers

The reason why tumor cells are adept at evading the recognition of antitumor immunity is related to their gene expression. Tumor cells and some immunosuppressive cells (including some bone marrow-derived suppressor cells, such as M2 macrophages) can highly express immunosuppressive signaling proteins (such as PD-L1 and CTLA-4) and bind to receptors on activated antitumor immune cells to achieve immunosuppressive effects [90]. Programmed cell death protein-1 (PD-1) is expressed in various cell types, including activated T cells, B cells, NK cells, and myeloid cells, and when it binds to programmed death-ligand 1 (PD-L1) or programmed death-ligand 2 (PD-L2) in tumor cells, this molecule can reduce the immune response [91]. Cytotoxic T lymphocyte-associated protein 4 (CTLA-4) acts as an inhibitory checkpoint for T cells and can bind to CD80/86 on DCs, inhibiting T cell activation and proliferation [92]. By restricting immune checkpoint pathways such as the PD-1/PD-L1 and CTLA-4 pathways, the immunosuppressive microenvironment induced by tumor cells can be effectively weakened. Based on this principle, immune checkpoint inhibitors (ICIs), such as nivolumab and pembrolizumab, have been developed in recent years, effectively contributing to cancer treatment [93].

In addition, adoptive cell therapies, especially chimeric antigen receptor (CAR)-T cell therapy, have flourished over the past decade, showing efficacy in immunotherapy for various nonsolid tumors [94]. CAR-T cell therapy is a gene editing technology that enables activated T cells to directly express a specific antigen recognition domain, namely, chimeric antigen receptor (CAR), to efficiently exert immune effects on tumor cells [95]. In addition, there are emerging therapies, such as CAR-NKs and CAR-Ms. Although basic and clinical research on the treatment of various solid tumors is advancing rapidly, the clinical treatment of solid tumors via adoptive cell therapy is still limited by the complex nature of the TIME.

Moreover, early screening and diagnosis with the help of cancer biomarkers is also an important application of the MCIB model in clinical oncology and tertiary prevention strategies, especially for the subtype of TIME. For head and neck squamous cell carcinoma (HNSCC), bioinformatics analysis based on The Cancer Genome Atlas (TCGA) and Gene Expression Omnibus (GEO) databases has helped researchers discover a variety of cancer biomarkers that affect TIME. Aminoacyl tRNA synthetase complex interacting multifunctional protein 1 (AIMP1) and cornichon family AMPA receptor auxiliary protein 4 (CNIH4) are significantly upregulated in HNSCC, in which AIMP1 expression levels are negatively correlated with immunity scores and microenvironment scores, and CNIH4 may affect the level of immune cell infiltration within the microenvironment and cancer immunotherapy [96,97]. Both AIMP1 and CNIH4 can be used as potential biomarkers for screening, diagnosis, and prognosis in patients with HNSCC. The transient receptor potential melastatin-subfamily member 7 (TRPM7) is a divalent cation-selective channel with a kinase domain [98]. Bioinformatics analysis using open databases showed that TRPM7 was negatively correlated with the expression of IL-6 receptor, TGF-β receptor and PD-L1 and negatively correlated with the level of immune-related cell invasion in cancers such as pancreatic adenocarcinoma and prostate adenocarcinoma [98].

## 6. Tumor Microbial Microenvironment

The tumor-associated microbiota is an important component of the TME for most cancer types, especially for the cancers of mucosal origin [99]. In addition to the existing microbiota near the tumor tissue, mucosal impairment caused by tumor growth and some components of the TME can also bring external microorganisms into the tumor tissue [100,101]. In addition, hematogenous transmission is an important way for microorganisms to colonize the TME [102]. Some microorganisms play key roles in tumorigenesis; for example, *Helicobacter pylori* is the main cause of gastric cancer, and hepatitis B virus (HBV) and hepatitis C virus (HCV) are important risk factors for hepatocellular carcinoma (HCC) [103,104]. In addition, other commensal microbial communities in the TME play important roles in tumorigenesis, progression, metastasis, and immune regulation.

### 6.1. Bacteria in the TBME

Bacterial infection can be regarded as an important environmental carcinogenic factor that can cause chronic infection before the tumor develops and induce the formation of a local inflammatory microenvironment. When histiocytes become cancerous, they cooccur in tumor tissues and participate in the development of the TME. Metabolites and lysates produced by partially endosymbiotic bacteria in tumor tissue may also accelerate tumor progression.

*H. pylori* in the gastric mucosa can produce virulence factors (such as VacA (vacuolating cytotoxin A) and CagA (cytotoxin-associated gene A)), induce oxidative stress in histiocytes (such as the production of reactive oxygen species (ROS) and reactive nitrogen species (RNS)), and promote normal biological activities (such as the decomposition of urea), leading to an increase in gastric pH and nitrosamine synthesis. These activities result in chronic inflammation and DNA damage in the gastric mucosa, which eventually leads to gastric cancer [105]. The composition of intestinal bacteria in the gastrointestinal tract can be altered by the influence of food, and the virulence factors produced by lipopolysaccharide (LPS) can induce inflammation, which plays an important role in the occurrence and progression of gastrointestinal tumors [106,107]. In addition, the effect of intestinal bacteria on tumors is not limited to the digestive tract, and these bacteria can also affect the occurrence and outcome of lung cancer [108]. Chaochao Xu et al. also showed that *Fusobacterium nucleatum* enrichment in the colorectal TME can induce M2 macrophage polarization and activate CCL20 [109]. This process can promote colorectal tumor metastasis by changing the post-metastatic niche outside the primary site [7,109].

### 6.2. Viruses in the TBME

Unlike those of bacteria, the viral tumorigenic mechanism and effects of viruses on tumor metastasis usually occur by mediating host cell DNA damage, targeting cell cycle-related signaling pathways, and inducing aberrant immune responses. Moreover, HBV can insert its own DNA sequence into the host cell genome, inducing mutations, which also promote the instability of the HBV genome, thus further increasing its ability to mediate insertional mutagenesis [110]. Long-term persistent necrosis of hepatocytes caused by chronic HBV infection, which induces an inflammatory response leading to cirrhosis, is also a key factor leading to HCC [111]. Notably, although HBV infection is an important risk factor for primary liver cancer, the rate of liver metastasis in patients with colorectal cancer (CRC) is significantly lower than that in patients without HBV infection [112]. This finding may be due to HBV affecting the hepatic TIME and interfering with the pri-metastatic niche, making it difficult for metastatic tumor cells to colonize the liver microenvironment [7,113].

## 7. Discussion

Based on the clinical combination treatment methods for tumors and previous research results on the temporal heterogeneity of the TME, we innovatively proposed the MCIB model to summarize the spatiotemporal heterogeneity of the TME. The model connects the four links of metabolic reprogramming, vascular remodeling, immune response, and microbial action in the process of tumorigenesis and progression in a spatial function, provides a microscopic picture of the TME in an orderly manner, and completely displays all the known components.

The four components of the MCIB model do not exist independently but intersect and show crosstalk with each other and play a role in tumorigenesis, progression, and metastasis. Among them, the crosstalk between the TMME and TCME, between the TMME and TIME, and between the TIME and TBME, especially between the TIME and TCME, are the most prominent. The hypoxic TME caused by tumor metabolic reprogramming is an important factor in stimulating tumor angiogenesis. The metabolic competition between tumor cells and immune cells and the interaction of metabolites in the TME can inhibit antitumor immunity. The expression levels of GLUT-1 and LDHA in tumor cells and the level of lactate in the TME are closely related to the antitumor immune effect of the body. A study of renal cell carcinoma showed significant reductions in CD8+ T cell infiltration and granzyme B and perforin levels in tumor tissues with high expression of GLUT-1 and LDHA [114]. Brand et al. reported that, compared with cells with low LDHA expression, melanoma cells with high LDHA expression had significantly reduced infiltration of activated CD8+ T cells and NK cells and significantly reduced levels of IFN-γ [115]. In addition to the effects of metabolic signatures, tumor-associated microorganisms inevitably undergo crosstalk when performing tumor immune functions on immune cells within the TME. For example, several oncolytic viruses can induce immunogenic cell death (ICD) in cancer cells and elicit innate and adaptive immune responses [116]. Cellular and animal experiments on measles virus (MV) and coxsackievirus B3 have validated the relevant immunological mechanisms [117,118]. In addition, bacterial peptides in melanoma can be presented by HLA molecules on antigen-presenting cells and melanoma cell membranes and play a role in shaping the melanoma TIME [119]. A study on the mycobiome in lung adenocarcinoma has shown that *Aspergillus sydowii* stimulates the production of the key molecule IL-1β through the b-glucan-mediated Dectin-1/CARD9 signaling pathway, thereby inducing MDSCs differentiation and promoting the formation of a tumor-suppressive TIME [120]. Furthermore, it is noteworthy that the mechanisms in TBME still need further investigation, such as the origin of microbiota in TME and its effects on tumor metabolism and angiogenesis, and the microbial lineage difference among the different types of cancers [99].

**Figure 2:**
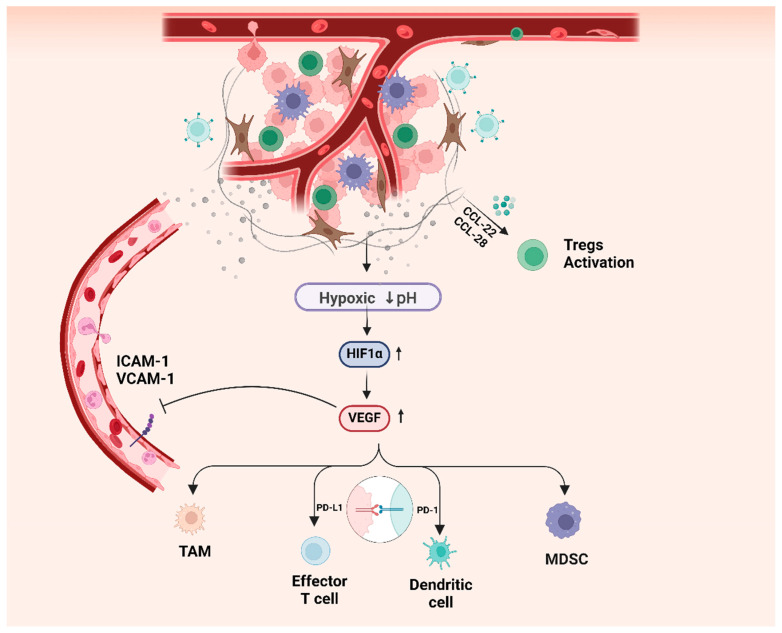
Crosstalk between the circulatory and immune microenvironment provides a theoretical basis for the combination of antiangiogenic therapy and immune checkpoint inhibitors. ICAM-1, intercellular adhesion molecule-1; VCAM-1, vascular cell adhesion molecule-1; TAM, tumor-associated macrophage; PD-1, programmed death-1; PD-L1, programmed death ligand-1; MDSC, myeloid-derived suppressor cell; HIF, hypoxia-inducible factor; VEGF, vascular endothelial growth factor; CCL, chemoattractant cytokine ligand. Figure created with BioRender.com (accessed on 30 January 2024).

In addition to the effects of tumor metabolism and microorganisms, the effects of tumor neovascularization on tumor immunity have also been widely studied and clinically applied. From an anatomical point of view, tumor neovascularization increases blood flow within tumor tissues and the extent to which tumor tissues are infiltrated by immune cells. However, the abnormal growth of tumor blood vessels and the expression of certain molecules may suppress antitumor immunity. An increased level of VEGF inhibits the expression of intercellular adhesion molecule-1 (ICAM-1) and vascular cell adhesion molecule-1 (VCAM-1), thereby inhibiting the adhesion of tumor-associated endothelial cells to leukocytes [121,122]. Abnormal vascular structure and reduced adhesion make it difficult for immune cells to reach tumor tissue through the vascular endothelium, hindering the infiltration of immune cells. VEGF also inhibits the NF-κB pathway induced by the proinflammatory factor TNF-α [123]. VEGFR is expressed in various immune cells, and VEGF can reduce effector T cell activity by directly binding to VEGFR1 or VEGFR2, mediating the infiltration of TAMs and MDSCs in tumor tissues, and increasing CD8+ T cells PD-1 and PD-L1 in DCs to inhibit their antitumor activity [124,125]. TAMs can also induce the recruitment of TECs by secreting proangiogenic substances such as VEGF and epidermal growth factor (EGF) [126,127]. The hypoxic TME also induces the upregulation of the chemokine ligands CCL-22 and CCL-28, thereby recruiting Tregs into tumors [128]. In addition, studies of glioblastoma have shown that IL-6 expressed by TECs can mediate the alternative activation of macrophages to produce M2-like macrophages with protumor effects [129]. The relationship between the TCME and the TIME is extensive and close, suggesting that the combination of antiangiogenic targeted drugs and immunotherapy is a new strategy for targeted therapy for tumors. This therapeutic strategy can enhance the effect of immunotherapy by normalizing tumor blood vessels and has been shown to be effective in several clinical trials [130]. For example, in the treatment of non-small cell lung cancer, the median progression-free survival (PFS) of the combination of the immune checkpoint inhibitor atezolizumab and bevacizumab based on the study IMpower150 was significantly longer than that of bevacizumab in combination with chemotherapy (8.3 months vs. 6.8 months, *p* < 0.001) [131]. In hepatocellular carcinoma, the median PFS was 6.8 months vs. 4.5 months (*p* < 0.0001) in the atezolizumab and bevacizumab combination group compared with the single-agent sorafenib group, which was significantly longer [132]. The mechanism of crosstalk between TCME and TIME is illustrated in Figure 2.

This article not only discusses the metabolism, microvascular, immunological, and microbial components in the TME, but also integrates them into an MCIB model according to the spatial dimension for the first time and comprehensively describes the mechanism of crosstalk between the spatial components. In fact, with the rapid development of bioinformatics in the past 30 years, many public databases and online platforms can provide empirical support for the components of TME in different cancer types and the crosstalk between the four subtypes. TCGA is an important database in tumor bioinformatics, providing comprehensive multi-omics data that has deepened our understanding of cancer genomics, molecular subtypes, and the TME. We present Table 2, which illustrates the assistance and adaptability of current bioinformatics data analysis to the MCIB model.

**Figure 3:**
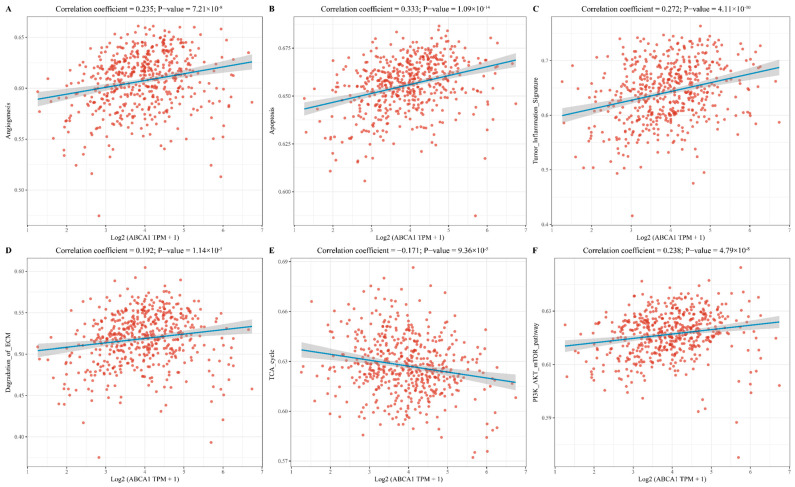
Spearman’s correlation analysis of the scores of 6 TME-related pathways and *ABCA-1* gene expression in LUAD. The abscissa represents the distribution of the gene expression, and the ordinate represents the distribution of the pathway score. Each red dot represents each patient’s *ABCA-1* score and the specific pathway score, the blue lines represent the regression results of Spearman’s correlation analysis, and the shades represent the 95% confidence interval. *ABCA-1* expression was positively correlated with (**A**) angiogenesis, (**B**) apoptosis, (**C**) tumor inflammation signature, (**D**) degradation of ECM, and (**F**) PI3K-AKT-mTOR_pathway, while negatively correlated with (**E**) TCA cycle. All the analysis methods and R packages were conducted in R version 4.0.3. *p*-Value < 0.05 was considered statistically significant.

Furthermore, we also employed the public databases to conduct bioinformatics analysis of the subtypes in the TME to verify the assistance of bioinformatics technologies for TME research. ATP-binding cassette subfamily A (*ABCA*) can mediate the transport of a variety of exogenous and endogenous substances across the lipid bilayer through ATP and is widely distributed in prokaryotes and eukaryotes [139,140]. In a previous study by Yanxia Yang et al., some members of *ABCA* were found to be associated with immune infiltration and *TP53* mutations in LUAD and could be used as a potential biomarker for the prognosis of LUAD, but *ABCA-1* was not included in the study [141]. Figure 3 presents the correlations between the *ABCA-1* gene and the scores of 6 TME-related pathways (including angiogenesis, apoptosis, tumor inflammation signature, degradation of ECM, TCA cycle, and PI3K-AKT-mTOR_pathway) in LUAD based on the TCGA database. We obtained RNAseq data (level 3) and corresponding clinical information of 516 samples for LUAD from the TCGA database (https://portal.gdc.cancer.gov/ (accessed on 11 September 2024)). The information about the *ABCA-1* gene that was contained in the corresponding pathways was collected, analyzed by the GSVA package of R 4.0.3, the parameter method = ‘ssgsea’ was selected, and finally, the correlation between genes and pathway scores was analyzed by Spearman’s correlation [142,143]. The expression of the *ABCA-1* gene was negatively correlated with TCA cycle and positively correlated with other signaling pathways, which visually and quantitatively demonstrates the correlation between *ABCA-1* and TMME, TCME, and TIME.

**Figure 4:**
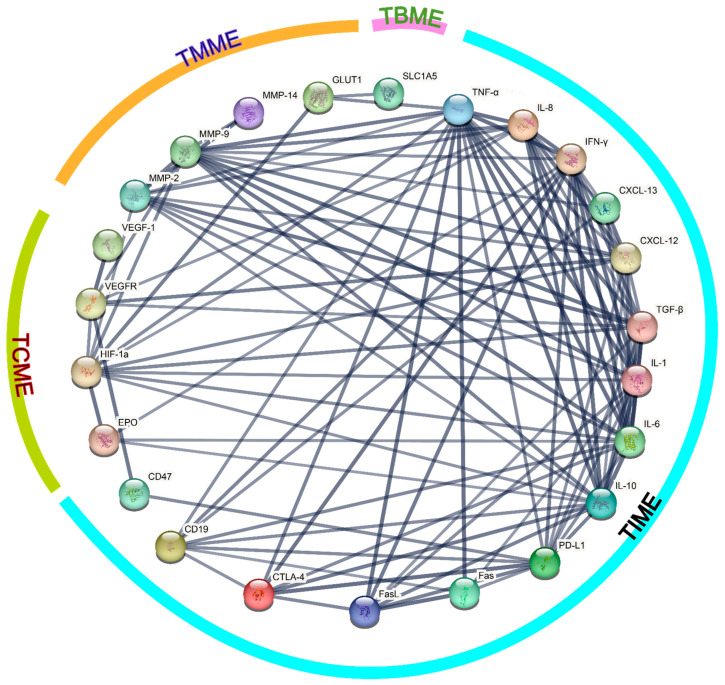
The protein-protein interaction network concerning 24 TME-related proteins.

Next, we imported the information of the TME-related proteins and genes mentioned in the paper into the STRING database (https://string-db.org/ (accessed on 10 September 2024)) to obtain the protein-protein interaction network diagram, which is shown in Figure 4. The minimum required interaction score was set to 0.700 (high confidence), from which 24 proteins were screened out, and the line thickness can indicate the strength of data support. The affiliation of each protein or gene among the four subtypes of TME has been indicated in the figure, and it can be concluded that there are extensive and close connections between the components of the various subtypes of TME, especially between TIME and TCME, and between TIME and TMME.

As mentioned in the introduction section, with the continuous advancement of various bioinformatics technologies and cancer biological databases, many mathematical modeling methods have been developed to characterize the deep landscape of the TME. In fact, as shown in Table 2, current research on TIME is at the forefront of other TME subtypes, and a large number of mathematical modeling methods have been developed to simulate TIME. The integration of multi-omics data, encompassing genomics, transcriptomics, proteomics, metabolomics, and spatial omics, offers a comprehensive approach to understanding the TME. By capturing the intricate interactions and heterogeneity within the TME, multi-omics data can significantly enhance the accuracy of existing modeling approaches, such as quantitative systems pharmacology (QSP) and agent-based models (ABM). For instance, Ruiz-Martinez et al. demonstrated the potential of coupling spatial ABM with whole-patient QSP models to simulate tumor growth and immunotherapy responses within the TME of triple-negative breast cancer [144]. Similarly, Nikfar et al. combined spatial QSP models and ABM to quantify intratumoral heterogeneity and immunoarchitecture [145]. On the basis of these studies, Zhang et al. integrated clinical trial spatial multi-omics analysis with virtual clinical trials to predict immunotherapy responses and discover biomarkers, which brings the application of spatial multi-omics modeling to clinical oncology [4]. Despite the promising advancements, challenges such as data integration and computational complexity remain. Future research should focus on developing robust analytical frameworks and leveraging AI and machine learning to analyze multi-omics datasets, paving the way for precision oncology.

Therefore, the simplified model proposed in this paper can provide a framework for cancer large-model analysis, the discovery of cancer biomarkers, the development of diagnostic reagents, and personalized treatment based on the MCIB model. For example, the MCIB model offers a novel framework for integrating bioinformatics analyses with comprehensive genomic databases like TCGA and GEO, facilitating the identification and validation of cancer biomarkers. By leveraging tools such as cBioPortal, QSP approaches, and STRING, researchers can explore the intricate interactions within the TME. Next, deep learning algorithms such as graph neural networks (GNNs) can be used to incorporate the component information of the TME into the model according to subtypes of the MCIB model. Then, information about non-tumor cells, metabolites, biomarkers, and signaling molecules in the TME, as well as their interrelationships, will be assigned to nodes and edges in the GNNs. Finally, considering the temporal heterogeneity of the tumor microenvironment during tumor progression, combined with the IEO model, a multi-dimensional dynamic TME interaction network based on cell biological behavior and intercellular crosstalk will be constructed, which will help us discover more tumor biomarkers and diagnostic/theragnostic agents. This model can be particularly influential for specific cancer types, with collaborative efforts between bioinformaticians, oncologists, and researchers. For example, in lung cancer, the TMME’s influence on tumor proliferation can be targeted, with biomarkers related to metabolic pathways being identified and validated. Metabolic inhibitors could also be evaluated in clinical trials based on biomarkers related to the TMME. Additionally, the impact of TBME on CRC underscores the model’s potential in identifying microbial biomarkers, which could lead to new therapeutic strategies. These applications of the MCIB model will provide approaches and ideas for solving the bottlenecks of TME and clinical oncology research mentioned above. In the next step, we will combine tumor intracellular signaling networks, MCIB model, and IEO model that describe the TME landscapes, as well as cancer clinical big data, multi-omics data, and AI to develop new methods for diagnosing and treating cancer under a multi-layered larger model.

## 8. Conclusions and Perspectives

There is the growing need for precision targeting of specific microenvironment subtypes through the development of novel therapies based on the TME heterogeneity. The MCIB model reaffirms the multiple subtypes of TME in spatial dimensions and explores innovative combination strategies that harness the synergies of tumor-targeted therapy, immunotherapy, anti-angiogenic therapy, and biotherapeutics to enhance the overall anti-tumor responses. Additionally, prioritizing the discovery and validation of robust biomarkers reflecting the spatiotemporal dynamics of the TME can guide tailored therapeutic interventions. The TME is spatiotemporally specific to different stages of tumor development. The MCIB model based on spatial functional heterogeneity is a theoretical supplement to the IEO model based on disease progression, and the combination of the MCIB model and the IEO model comprehensively outlines the spatiotemporal evolution of the TME. This study provides a theoretical framework for the development of targeted drugs targeting different microenvironment subtypes and for the combination of tumor-targeted therapy and immunotherapy according to the crosstalk of different subtypes of the microenvironment. According to the theoretical paradigm of this study, the mathematical logic-based methods and AI methods will be integrated into the model for computation, which in turn provides a comprehensive spatiotemporal analysis of tumor origin, growth evolution, and metastasis. Furthermore, this study presents a new research direction for the treatment of tumors, elucidating the complex biological behavior of tumors and clinical oncology, and bridging the gap between basic oncology and clinical oncology. These directions aim to advance the understanding of tumor biology and ultimately improve personalized approaches to cancer treatment.

## Figures and Tables

**Table 1:** The characteristics of the MCIB model.

Characteristics	TMME	TCME	TIME	TBME
Definition	The interaction and regulation process between metabolites, metabolic enzymes, and metabolic pathways in tumor cells.	A range of components of the blood circulation that interact with tumor cells.	Immune cells and molecules in TME that interact with tumor cells and are involved in tumor control or evade immune surveillance.	Microbial communities in tumor tissues and the ecological environment associated with microorganisms.
Components	CAFs, ECM, lactic acid, glucose, ROS, RNS, CO_2_, O_2_	TECs, pericytes, RBC, PLT, O_2_, VEGF, HIFs	CD8+ T cell, CD4+ T cell, Tregs, NK cell, TAMs, DCs, B cell, MDSCs, granulocytes	*Helicobacter pylori*, *Fusobacterium nucleatum*, HBV, Oncolytic virus, etc.
Relative treatment strategies	Metabolism-interference therapy	Anti-angiogenesis therapy	Immunotherapy	Antibiotic and antiviral drugs
Drug targets	LDHA targeted drugs, MCT-1 targeted drugs, GLS targeted drugs	VEGF targeted drugs, HIF-1α targeted drugs	Immune checkpoint inhibitors, adoptive cell therapy	None

CAFs, cancer-associated fibroblasts; ECM, extracellular matrix; ROS, reactive oxygen species; RNS, reactive nitrogen species; TECs, tumor-associated endothelial cells; PLT, platelet; VEGF, vascular endothelial growth factor; HIFs, hypoxia-inducible factors; Tregs, regulatory T cells; TAMs, tumor-associated macrophages; DCs, dendritic cells; MDSCs, myeloid-derived suppressor cells; HBV, hepatitis B virus.

**Table 2:** The bioinformatics analysis based on TCGA in TME research.

TME Subtype	Cancer Type	Subject of the Study	Bioinformatic Approaches	Effects of Subjects	Reference
TMME	Lung adenocarcinoma (LUAD)	LINC01614 (CAFs-specific lncRNA)	Microarray technology, metabolomics analysis, differential expression analysis, gene set enrichment analysis	Exosome-packaged lncRNA LINC01614 produced by CAFs can mediate enhanced glutamine uptake in LUAD cells, which in turn affects TMME through tumor metabolic reprogramming.	[133]
TCME	13 cancer types including breast cancer and laryngeal squamous cell carcinoma, etc.	Endothelial Myct1	Expression profiles analysis (Pearson’s chi-square test), GSVA, transcriptomic analysis	As an inhibitory regulator of tumor angiogenesis, Myct1 can affect the production and regulation of TCME and TIME.	[134]
TIME	Stomach adenocarcinoma (STAD)	Expression profiles of 37 T cell-related genes	DEG identification, KEGG enrichment analysis, construction of prognostic signature (LASSO regression, Cox regression), validation of external cohort (K-M analysis)	T cell regulatory factors SERPINE2 and CXCL12 promote the exhaustion of T cells and proliferation of STAD cells.	[135]
	Lung squamous cell carcinoma (LUSC)	494 samples of the Illumina HiSeq 2000 gene expression data	Analysis of cell proportion (ssGSEA algorithm), analysis of sample subtypes (K-M analysis), DEG enrichment analysis, interaction networks analysis, validation of hub genes	Immune checkpoint genes such as CD47 and SILPA, as well as hub genes such as CD19 and CTLA-4, can be used as biomarkers to help identify the immune microenvironment of LUSC.	[136]
	Kidney renal clear cell carcinoma (KIRC)	12 cuproptosis genes	GSVA, survival associations analysis, clustering analysis (NMF method, LASSO regression), DEG enrichment analysis	Cuproptosis may affect the immune microenvironment of KIRC and can be used as a predictive biomarker for immunotherapy.	[137]
TBME	HBV-related hepatocellular carcinoma (HCC)	Ferroptosis-related gene SLC1A5	Survival analysis (K-M analysis), prognostic value evaluation (Cox regression), Spearman’s correlation analysis, GSVA	SLC1A5 is associated with the immunosuppressive TME of HBV-associated HCC and may serve as a biomarker for HBV-associated HCC.	[138]

lncRNA, long noncoding RNAs; GSVA, gene set variation analysis; DEG, differentially expressed genes; KEGG, Kyoto Encyclopedia of Genes and Genomes; ssGSEA, single-sample gene set enrichment analysis; K-M analysis, Kaplan–Meier analysis; NMF, non-negative matrix factorization.

## Data Availability

Data sharing is not applicable to this article, as no new data were created or analyzed in this study.
